# Target Characterization of Kaempferol against Myocardial Infarction Using Novel In Silico Docking and DARTS Prediction Strategy

**DOI:** 10.3390/ijms222312908

**Published:** 2021-11-29

**Authors:** Xunxun Wu, Xiaokun Li, Chunxue Yang, Yong Diao

**Affiliations:** 1 School of Biomedical Science, Huaqiao University, Quanzhou 362021, China; wuxunxun2015@163.com (X.W.); 18014071010@stu.hqu.edu.cn (X.L.); 2 Department of Pathology, The University of Hong Kong, Hong Kong 999077, China

**Keywords:** target identification, kaempferol, docking, DARTS, Src

## Abstract

Target identification is a crucial process for advancing natural products and drug leads development, which is often the most challenging and time-consuming step. However, the putative biological targets of natural products obtained from traditional prediction studies are also informatively redundant. Thus, how to precisely identify the target of natural products is still one of the major challenges. Given the shortcomings of current target identification methodologies, herein, a novel in silico docking and DARTS prediction strategy was proposed. Concretely, the possible molecular weight was detected by DARTS method through examining the protected band in SDS-PAGE. Then, the potential targets were obtained from screening and identification through the PharmMapper Server and TargetHunter method. In addition, the candidate target Src was further validated by surface plasmon resonance assay, and the anti-apoptosis effects of kaempferol against myocardial infarction were further confirmed by in vitro and in vivo assays. Collectively, these results demonstrated that the integrated strategy could efficiently characterize the targets, which may shed a new light on target identification of natural products.

## 1. Introduction

Natural products have historically served as a prolific and unsurpassed source for novel candidates in the search for new drugs [1]. Target identification of known bioactive compounds and novel analogs is pivotal to understanding the therapeutic effects and underlying mechanisms of natural products [2]. How to identify the therapeutic target from the huge number of compounds from natural products is a challenging and costly task. Even though the identification and validation strategies have been improved, such as affinity chromatography of immobilized probe, label-free methods including drug affinity responsive target stability (DARTS), and virtual screening techniques. However, limitations still exist in the progress of single-method target identification, such as target selection and lower-abundance targets identification [3,4].

DARTS is a robust method for the determination of target proteins for natural products without chemical modification. The concept of DARTS is that ligand-bound proteins show altered stability compared to ligand-unbound proteins in the case of proteolysis. However, the DARTS method is not sensitive in identifying low-abundance proteins and validating of proteolysis of a cell lysate [2,3]. In silico target identification could be performed independent of target abundance and could be a useful complement for traditional bench experiments [5]. Chemical similarity is a key criterion for in silico target identification. Structurally similar compounds have similar physicochemical properties and can show possibly similar biological effects [6,7,8]. Based on the similarity to a biologically active template, the structurally similar strategy offers an alternative avenue for the exploration of ligand–target interactions with a high chance and hit rate [9,10]. As the saying goes: ‘The best way to discover a new drug is to start with an old one’, which demonstrates that conventional drugs or targets may have new uses [11].

The Src tyrosine kinase is a non-receptor tyrosine kinase, and is widely discussed as a factor in tumorigenesis through regulating cell growth, differentiation, adhesion, and survival [12]. In addition, inhibition of Src ameliorates myocardial ischemia reperfusion injury and arrhythmia [13,14]. This evidence raises the possibility that the regulation of Src could relieve myocardial infarction. However, Src inhibitors, such as dasatinib, caused coronary artery disease in the clinic [15]. Therefore, it is important to discover the regulatory mechanism of Src and develop safe and effective inhibitors. Kaempferol (Kae) is one of the most commonly used flavonoids from natural products, such as Ginkgo biloba. Kae has a variety of therapeutic effects, for example, anti-inflammatory, anticancer, and antibacterial properties [16]. In addition, recent studies demonstrated that Kae could protect rat brain against I/R-induced damage [17,18]. However, the specific target and molecular mechanism remain to be identified.

**Figure 1:**
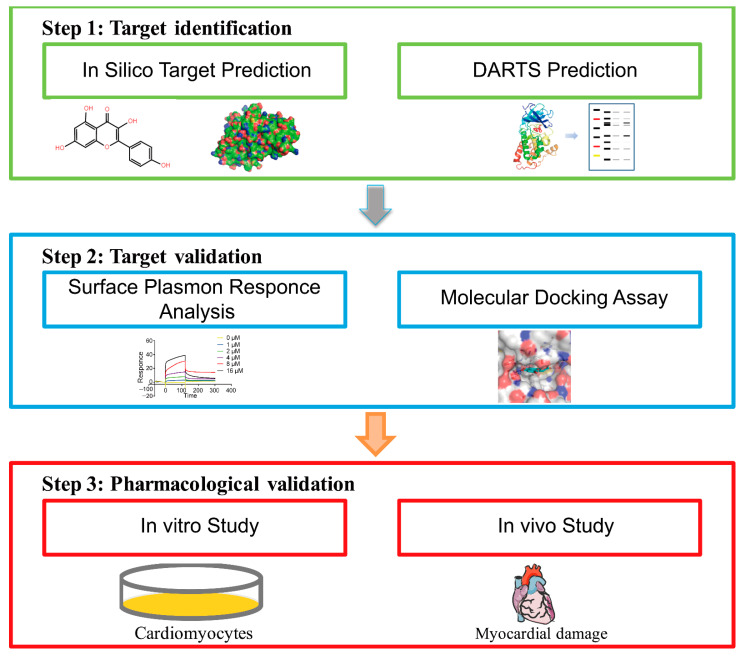
The flow diagram of target characterization of Kae against myocardial infarction using comprehensive in silico docking and DARTS prediction strategy.

In this study, we proposed a novel strategy that combined the in silico target identification and DARTS prediction method to identify the direct target of Kae. Then, compound-receptor interactions were further confirmed by molecular docking and surface plasmon resonance (SPR) analysis. Furthermore, the cardioprotective effects against myocardial infarction were analyzed (Figure 1). This initial phase of target identification can be done rapidly by integrated approaches to identify the optimal lead from different structural isomers. This novel methodology may help uncover new therapeutic targets and molecular pathways for myocardial infarction therapy. In addition, this knowledge could shed light on identifying new therapeutic targets and molecular pathways for currently untreatable diseases.

## 2. Results

### 2.1. Target Identification of Kae by In Silico Docking and DARTS Prediction Strategy

**Figure 2:**
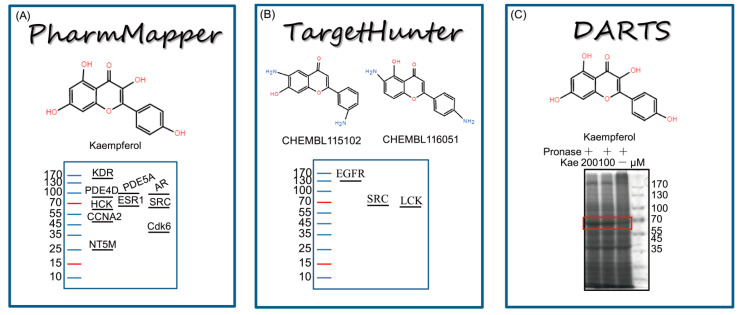
(**A**) The ranked list of hit targets of Kae from PharmMapper. (**B**) Target identification of Kae by TargetHunter using the 2D similarity compounds. (**C**) The DARTS assay was employed to detect the different bands, and a ~60 kD band increased upon Kae incubation. The red box indicated the detected bands at ~60 kD.

The drug affinity responsive target stability (DARTS) approach is widely used for direct target protein identification. To reveal the direct target of Kae in cardioprotection, DARTS method was employed to find the potential candidates that could interact with Kae. Of particular note, to ensure the binding-promoted stability efficiency, the concentration of Kae used for DARTS assay was higher than that used in cell culture. Thus, after proteolytic digestion, high concentrations of Kae (100 and 200 μM) were added to H9C2 cells lysates and incubated for 1 h, respectively. Coomassie-blue-stained results showed that the abundance of the band at ~60 kDa was increased after the incubation with Kae, which may be caused by the resistance to pronase degradation (Figure 2C). PharmMapper is a web server for potential drug target identification with a comprehensive target pharmacophore database. On the other hand, TargetHunter is designed to search for target identification based on chemical similarity. In this study, PharmMapper Server and TargetHunter [19] tools were adopted for potential drug target identification (Figure 2A,B). The docking results obtained from PharmMapper were further ranked by z-score (Supplementary Table S1). From the top 10 target candidates of Kae identified by PharmMapper, tyrosine-protein kinase HCK and proto-oncogene tyrosine-protein kinase Src are ~60 kDa (Table 1). In addition, potential drug targets obtained from TargetHunter were ranked by the similarity to Kae (Supplementary Table S2). Tyrosine-protein kinase LCK, epidermal growth factor receptor erbB1, and tyrosine-protein kinase Src are published targets of compounds CHEMBL 115102 and CHEMBL116051, which are similar to Kae [20]. Among the three targets, tyrosine-protein kinase LCK and tyrosine-protein kinase Src are ~60 kDa (Table 2). Collectively, tyrosine-protein kinase Src has been identified as the best potential target of Kae through the integrated screening of DARTS and in silico target identification.

### 2.2. Src Is a Direct Target of Kae

**Figure 3:**
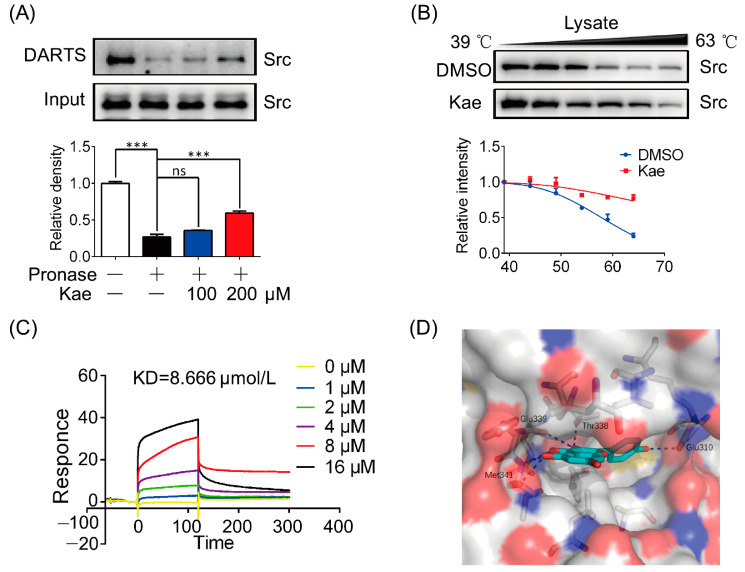
Identification and validation of Src as a direct target for Kae. (**A**) Immunoblot analysis of Src treated with DARTS assay (*n* = 3). (**B**) Immunoblot analysis of Src degradation insult by indicated temperature (*n* = 3)**.** (**C**) Surface plasmon resonance (SPR) analysis of the interaction between Kae and Src: KD = 8.666 μmol/L (*n* = 3). (**D**) Predicted binding mode of Kae in the ATP-site of the Src catalytic domain. Protein is shown in surface, and key amino acids interacting with Kae are highlighted in stick representation. Kae is colored in cyan. Hydrogen bonds are depicted by blue dashed lines. *** *p* < 0.001 and ns, statistically not significant.

To validate whether Kae was directly bound to Src, pronase was added to cell lysate and incubated with Kae. The results showed that Kae increased the resistance to pronase degradation and also promoted the stability of Src (Figure 3A). To further examine the interaction between Kae and Src, a CETSA assay was performed. As shown in Figure 3B, the addition of Kae to heat-denatured H9C2 cell lysates led to the stabilization of Src at different temperatures. Surface plasmon resonance (SPR), as a powerful technique, has been widely employed for the detection of protein–probe interactions [21]. SPR analysis revealed the potential interaction between Kae and Src (K_D_ = 8.666 μM) (Figure 3C). As predicted by molecular docking, there were several hydrogen bonds formed between Kae and the hinge region of Glu339 and Met341, as well as the gatekeeper residue of Thr338. H-bonding interactions are typical characteristics for the binding of ATP-competitive inhibitors with kinases [22]. The phenol moiety of Kae extends into the ATP back-site and makes one hydrogen bond with Glu310. In addition to polar interactions, the aromatic rings of Kae establish hydrophobic contact with lipophilic residues present in the ATP-site of the Src catalytic domain (Figure 3D). Collectively, these data suggest that Src is a direct target of Kae.

### 2.3. Kae Protects Cardiomyocytes against Oxidative Damage

**Figure 4:**
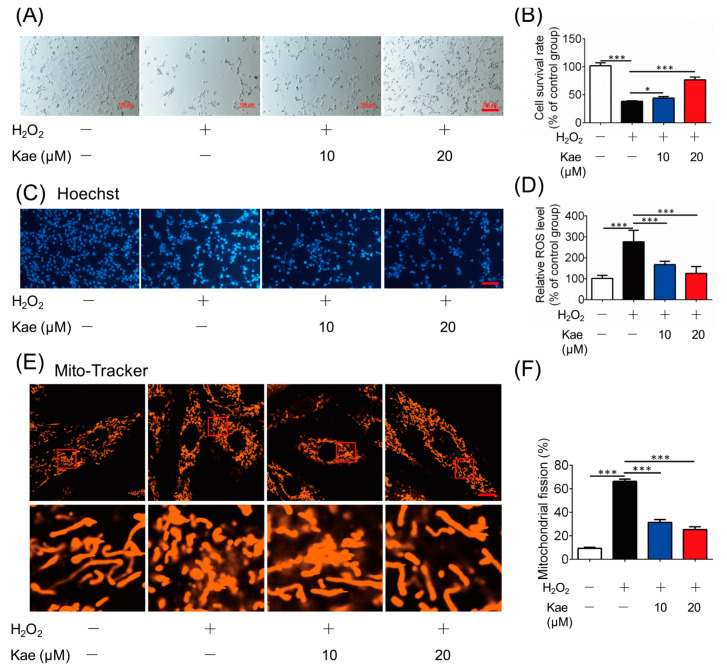
Kae protects cardiomyocytes. (**A**) Cell morphology analysis (scale bar, 100 µm). (**B**) Cell survival rate analysis (*n* = 6). (**C**) Kae-induced nuclear morphological changes were examined by using fluorescence (scale bar, 100 µm). (**D**) Intracellular ROS production (*n* = 6). (**E**) Mitochondrial fission imaging. (Scale bar, 5 μm). (**F**) Quantification of relative mitochondrial fission ratio (*n* = 6). * *p* < 0.05, *** *p* < 0.001.

To further verify the protective effects of Kae, we applied cardiac myoblast H9C2 cells for in vitro assays. H9C2 cells were maintained in medium supplied with different concentrations of Kae (10 and 20 μM). The results showed that the survival rate was increased with the addition of Kae, indicating that Kae protected cell survival against H_2_O_2_ insult (Figure 4A,B). Hoechst 33342 is an apoptotizing cell detection fluorescent probe that brightly stains chromatin in cell nuclei [23]. Apoptotizing cells exhibit apoptosis-related alterations in the chromatin state. In the H_2_O_2_ treated group, the nucleus showed extremely bright zones, which suggested abnormalities in the nucleus size or shape, and heterogeneous staining of chromatin. However, the cells treated with Kae were homogenously stained with Hoechst 33342, indicating that the addition of Kae protected the nucleus from oxidative damage (Figure 4C). Oxidative stress is a significant characteristic in myocardial infarction, and Kae treatment effectively suppressed H_2_O_2_-induced ROS generation (Figure 4D). In addition, the ROS level was detected, and the ROS level was reversed by the treatment of Kae, suggesting that Kae prevented mitochondrial fragmentation through the inhibition of oxidative stress (Figure 3E,F). In conclusion, these results indicate that Kae could protect against and prevent cell apoptosis via mitochondria protection.

### 2.4. Kae Protects the Heart against Ischemic Injury

**Figure 5:**
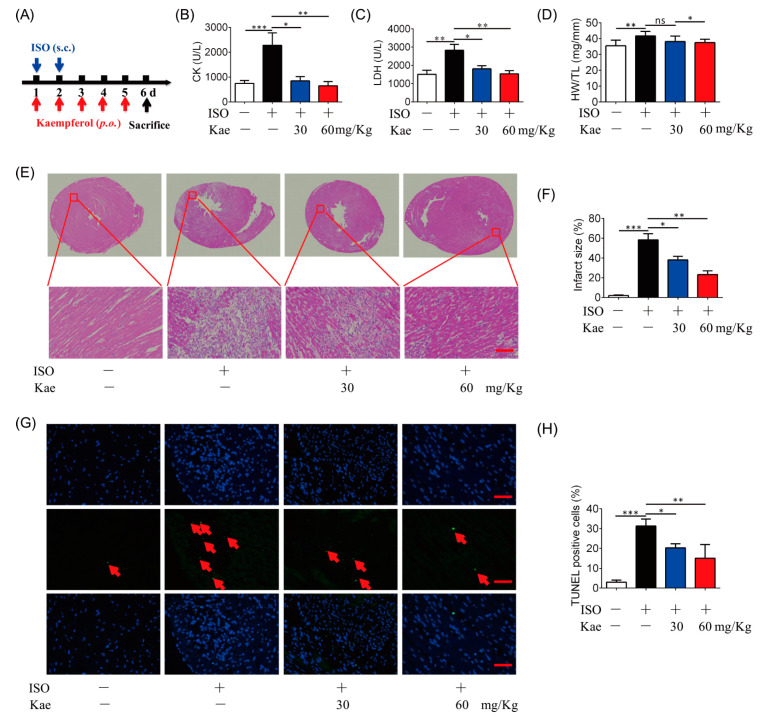
Kae protects the heart from ISO-induced myocardial damage in rats. (**A**) The myocardial damage in rats was established with isoprenaline (ISO) for 2 days, and Kae was orally administrated (30, 60 mg/kg, 5d). (**B**) The level of CK in the blood (*n* = 6). (**C**) The leakage of LDH in the blood (*n* = 6). (**D**) The ratio of heart weight to tibia length (HW/TL) (*n* = 6). (**E**) Representative HE-stained heart sections, the lower panel are enlarged from the red boxex in the upper panel (*n* = 6, scale bar, 50 µm). (**F**) Quantitative analysis of infarct size. (**G**) Apoptosis detection using TUNEL staining assay (scale bar, 50 µm). The red arrows indicate TUNEL positive cells. (**H**) Quantification of TUNEL positive cells (*n* = 6). * *p* < 0.05, ** *p* < 0.01, *** *p* < 0.001 and ns, statistically not significant.

To further examine the effects of Kae on cardioprotection, isoprenaline (ISO)-challenge-induced heart injury animal model was established [24] (Figure 5A). ISO treatment increase the leakage of creatine kinase (CK) and lactate dehydrogenase (LDH) in the rat blood, which was rescued by oral administration of Kae (30, 60 mg/kg) (Figure 5B,C). HE staining of heart tissue demonstrated that ISO induced cardiac injury with the color, texture, the presence of scar tissue and areas of softening or discoloration of septum, and broken fiber, but Kae administration reduced the cardiac infarct surface with structure normalization (Figure 5E,F). In addition, the ratio of heart weight and tibial length was increased by ISO insult, and oral administration of Kae at 60 mg/kg decreased the ratio (Figure 5D). Nuclear chromatin fragmentation is a hallmark apoptosis, which leads to an appearance of broken DNA strands [25]. We have applied terminal transferase dUTP nick-end labeling (TUNEL) assay to detect DNA degradation, which reveals a percentage of apoptosis cells. We found Kae treatment decreased the apoptosis cells to prevent cardiomyocyte apoptosis in mouse heart challenged with ISO (Figure 5G,H) compared with the control group. Therefore, Kae could alleviate heart injury induced by ISO via anti-apoptosis pathway.

## 3. Discussion

Target identification is used to study all binding targets that account for biological effects. Despite the technological advances in natural products development, the majority of their putative biological targets remain unknown [26,27]. In recent years, extensive strategies have been presented on target identification, such as affinity-based protein profiling, “label-free” methodology, and in silico docking [2,3,28]. However, these methods provide large numbers of candidate targets in a single experiment, which present us a new challenge for target selection. In the present study, a novel strategy that integrated of in silico docking and DARTS prediction was established, and Kae was selected as an example for investigation. With this strategy, the probable mass of the potential target can be identified by DARTS method, and in silico docking study can further help to predict the candidates. Therefore, using this strategy, Src protein was rapidly focused on as one of the candidate targets of Kae, in silico analogue, and pharmacophore docking. In addition, DARTS, CETSA, and SPR assays further identified that Kae was directly bound to Src. In vitro and in vivo study demonstrated that the cardioprotective effect of Kae was through anti-apoptosis pathway.

Traditionally, mass-spectrometry-based analysis would be employed followed by DARTS screen to further determine the candidate proteins of the specific gel band. However, the mass spectrometry data was informatively redundant for identifying the direct target of Kae. Recently, a large number of in silico target identification methods have been used and studied through open-source web servers, which are relatively fast and convenient, while it is still a challenge to reduce the number of false positives [29]. Therefore, the improvement of target identification strategies with high accuracy of target information is particularly important. Herein, a comprehensive in silico target identification and DARTS prediction strategy was established, which could quickly identify targets from a complex potential target pool.

The PharmMapper online tool is a convenient web server for potential drug target identification by reversed pharmacophore matching. Through target fishing by PharmMapper Server, Src was ranked the highest. TargetHunter is an in silico target identification tool for predicting targets based on chemical similarity searching, which is easy to operate and has high accuracy. When the structure of Kae was submitted to TargetHunter, CHEMBL115102 and CHEMBL116051 were identified as structurally similar compounds to Kae, and they share the target of Src. Nevertheless, the limits of both target identification methods were proven to be at least partially circumvented when the two different strategies were used in combination with each other. Indeed, several bands seemed to increase with Kae, such as the band located at ~60 kDa and 170 kDa. TargetHunter and PharmMapper methods are available online, and are free, fast, and convenient. However, compared to commercial software, the limited available database is the main obstacle ahead. In this study, TargetHunter and PharmMapper methods were performed, and a small number of targets with high mass are included in the database. Herein, the targets at 170 kDa were not top-ranked from the in silico study; thus, the band located at ~60 kDa was selected as an example to research. However, with the technological advances in machine learning and artificial intelligence methods, more comprehensive database and new methods for target identification are still on the way. Therefore, a constant improvement of target identification strategies is required for achieving more efficient and reliable targets.

Natural products have involved a large number of structural isomers and analogue compounds, with multiple structural isomers involved in different biological activities [30]. It was reported that the similar structures of compounds would tend to show similar biological characteristics and effects, and similar molecules are efficient for lead optimization [31]. With increasing knowledge of receptor–ligand interactions, the prediction method by similarity search is quite meaningful [31]. In recent years, the concept of molecular similarity has grown dramatically in the area of target identification. In addition, structure similarity search methods are especially applicable in natural products searches and useful in molecular mechanism elucidation [9]. Kae, a flavonoid, is structurally similar to other flavonoids. Thus, Kae was selected as a case study.

Myocardial infarction is a major cause of death in modern society, and Src family protein tyrosine kinase has been identified as a promising target for treating cardiovascular diseases, such as hypertension and ischemic heart disease [13]. NaKtide, a Na/K-ATPase-derived peptide Src inhibitor, ameliorates myocardial ischemia-reperfusion injury in vitro and in vivo [14]. In addition, Src inhibition improves arrhythmia through reducing the internalization and degradation of connexin 43 in the heart [32]. Interestingly, mitochondrial Src tyrosine kinase is inhibited by H/R from rat hearts. Inhibition of mitochondrial JNK/Sab/Src/ROS pathway could ameliorate H/R-associated oxidative stress [33]. However, dasatinib, a first-phase anti-acute myeloid neoplasms drug, leads a side effectof coronary artery disease [15]. Therefore, it is crucial to discover the endogenous cardiac arrhythmias regulatory mechanism of Src and develop safe and effective inhibitors. Kae, an ingredient isolated from natural products, has revealed a cardioprotective effect against myocardial infarction. Herein, using our novel strategy, the cardioprotective target of Kae was identified and verified.

However, several limitations still exist in this study. ISO-challenge-induced heart damage provides an easily operated model which produces myocardial damage similar to that seen in acute cardiac ischemia in humans [34]. To further evaluate the cardioprotective function of Kae on myocardial infarction, a coronary-artery-ligation-induced myocardial infarction model may be performed. In addition, creatine kinase (CK) and lactate dehydrogenase (LDH) were not specific to heart injury, and detection of CK-MB or troponin (T/I) in the blood might be a better choice. Echocardiography is the primary imaging modality for detecting cardiac functions. In short, further results about cardiac function require further study.

## 4. Materials and Methods

### 4.1. Reagents

Kaempferol (purity ≥98%) was obtained from Chengdu Biopurify Phytochemicals Ltd. (Chengdu, China). Mito-Tracker was obtained from Thermo Fisher Scientific (Xiamen, China). Isoprenaline hydrochloride (#I5627) was purchased from Sigma (St. Louis, MO, USA). Antibody against Src (#36D10) Rabbit mAb (#2109) was purchased from Cell Signaling Technology (Beverly, MA, USA). RIPA lysis buffer (#P0013D) and BCA assay (#P0010) were obtained from Beyotime (Suzhou, China).

### 4.2. Identification of Candidate Targets of Kae

The structure of Kae was drawn by ChemBio3D Ultra 14.0 software, and the mol2 format structure of Kae was uploaded. Then, results were presented after completing screening and scoring protocol for each target set by PharmMapper software [35] (http://www.lilab-ecust.cn/pharmmapper/, accessed date 2 October 2021). In addition, the Kae pharmacological targets were pooled with TargetHunter [19] (http://www.cbligand.org/TargetHunter, accessed date 2 October 2021).

### 4.3. Animals and Treatments

Sprague Dawley (SD) rats (Male, 200–220 g) were purchased from Wushi Animal Center (Fuzhou, China). All animal experiments were carried out in accordance with the National Institutes of Health guide for the care and use of laboratory animals, following protocols approved by ethics committee of Huaqiao University (no: A2020033). The mice were housed in cages with a constant temperature (20 ± 2 °C) and a 12-h light/dark cycle with free access to standard food and water.

The myocardial damage model on rats was established by ISO (65 mg/kg, *s.c.*, 2 days), as described previously [36], and Kae (30, 60 mg/kg, *p.o.*) was administrated for 5 consecutive days. Then, rats were euthanized, and the heart tissues were collected for further analysis.

### 4.4. Cell Culture

H9C2 cells were obtained from Cell Bank of Chinese Academy of Sciences and cultured in the medium of DMEM supplemented with 10% (*v*/*v*) FBS in an incubator with conditions of 37 °C with 5% CO_2_ in air atmosphere.

For cell survival assay, H9C2 cells were seeded in a 96-well plate and incubated with Kae at given concentrations for 8 h with H_2_O_2_ (100 μM). The cells were observed by bright-field microscopy to determine the cytotoxicity. Cell survival was evaluated by Cell Counting Kit-8 (CCK8, APExBIO, Shanghai, China).

### 4.5. Immunoblotting Experiments

Cell lysates were harvested with RIPA lysis buffer, and the protein quantitation of all the samples was performed using BCA assay. Then, samples were separated by SDS-PAGE and transferred onto PVDF (0.45 μm) membrane. PVDF membranes were incubated with indicated primary antibody overnight at 4 °C followed by blocking for 1 h. The next day, membranes were washed and incubated with secondary antibodies for 2 h at room temperature. The protein bands were imaged using Tanon 500 system (Tanon, Shanghai, China).

### 4.6. Surface Plasmon Resonance (SPR) Analysis

Src protein was immobilized on a carboxymethylated 5 (CM5) sensor chip. Different concentrations of Src (1 to 16 μmol/L) were used for analysis using the Biacore T200 system (GE Healthcare Life Sciences, Uppsala, Sweden).

### 4.7. Molecular Docking

The 3D structure of the Src kinase catalytic domain in complex with the drug bosutinib was downloaded from the Protein Data Bank (PDB entry 4MXO). Upon removing bosutinib and water molecules, hydrogen atoms were added to the protein according to the protonation states of chemical groups at the physiological pH. The initial 3D conformer of Kae was generated using the ETKDG method implemented in RDKit (version 2020.09), and further minimized with MMFF94s force field. Kae was docked into the ATP site of the Src catalytic domain by the program LeDock [37].

### 4.8. The Assay of Mitochondrial Fission

After treatment, H9C2 cells were incubated with 50 nmol/L Mito Tracker for 30 min at 37 °C. Then, after washing with warm PBS three times, the mitochondrial fission was determined on confocal scanning microscopy (Zeiss, LSM 700).

For quantification of mitochondrial fission, the fluorescence images were performed using ImageJ software, as described previously [38]. Briefly, appropriate threshold for images were set up, and individual mitochondrion were analyzed for circularity (4π × area/perimeter^2^) and lengths of major and minor axes. The form factor (FF, the reciprocal of circularity value) and aspect ratio (AR, major axis/minor axis) were calculated. While the mitochondrion was a small perfect circle, parameters have a small value, and the values increase when it becomes elongated. The lower values of FF and AR indicate mitochondrial fission. In addition, fragmented or tubular mitochondria were counted by three experimenters.

### 4.9. Drug Affinity Responsive Target Stabilization Assay (DARTS)

The DARTS assay was conducted in H9C2 cells. In brief, approximately 1 × 10^7^ cells were lysed on ice for 30 min. Indicated concentrations of Kae (diluted in 1 × TNC buffer, 50 mmol/L Tris, 50 mmol/L NaCl, 10 mmol/L CaCl_2_, pH = 7.4) were added into the aliquoted protein (5 mg/mL). Then, samples were gently mixed and incubated for 2 h at room temperature. Then, lysates were digested by pronase (1:400, *w*/*w*) for 30 min. Then, 1 × loading buffer was added and boiled for 10 min. The samples were separated by sodium dodecyl sulfate polyacrylamide gel electrophoresis (SDS-PAGE) and stained with Coomassie blue.

### 4.10. Cellular Thermal Shift Assay (CETSA)

For cell lysate CETSA experiments, H9C2 cells were lysed with a freeze-thawed method using liquid nitrogen. Then, cell lysates were divided into two fractions, one incubated with the DMSO as the control group and the other incubated with Kae (200 μmol/L) for 30 min as the Kae-treated group (at room temperature). Then, the lysates from the two groups were aliquoted, respectively, followed by heating at sequentially increased temperature (39–63 ℃ with a 4 ℃ interval) for 5 min. After boiling for 10 min, immunoblotting assay was performed, and Src abundance level was analyzed.

### 4.11. Hoechst 33342 Staining

Cells were treated and harvested, followed by rapid staining, and fixed in 4% paraformaldehyde for 20 min at room temperature. Then, cells were washed with PBS for 5 min. After incubation with Hoechst 33342 (100 ng/mL) in the dark for 15 min, the cells were viewed using a fluorescence microscope.

### 4.12. Tissue Preparation, Hematoxylin Eosin and TUNEL Staining

First, tissue samples were fixated with 4% paraformaldehyde for 24 h, then also with cellular water. Graded alcohols (70%, 80%, 95%, and 100%) were used in dehydration for 1 h each. Next, 100% xylene was used for tissue clearing (1 h). After clearing, tissue sections were infiltrated with paraffin wax to support the tissue for thin sectioning. 5 μm thick heart cross-sections were cut, and the paraffin wax was removed, followed by tissue staining. Six serial cross-sections were collected and then placed in slide boxes and stored until use.

For H&E staining, the hematoxylin solution stains the nuclear chromatin and possibly other acidic cellular elements. Unbound hematoxylin is removed with water rinses, followed by an optional differentiation step using acid alcohol. Staining was observed using a light microscope.

TUNEL staining was performed using a One-Step TUNEL Apoptosis Assay Kit (Cat# C1086; Beyotime, Suzhou, China) according to the manufacturer’s protocols with modifications. First, cross-sections were incubated with proteinase K (20 μg/mL) for 15 min. Following digestion, TUNEL reaction mix was added onto the slides and incubated at 37 ℃ for 1 h. After washing, staining was observed using a light microscope. All digital images were captured using a Nikon digital camera.

### 4.13. Determination of LDH and CK Leakage

Blood samples collected from mice were centrifuged at 3000× *g* for 30 min to obtain serum. LDH and CK leakage was determined by a colorimetric procedure with lactate dehydrogenase assay kit (A020-2-2) and creatine kinase assay kit (A032-1-1, Nanjing Jiancheng Bioengineering Institute, Nanjing, China) according to manufacturer’s instructions, and the absorbance at 490 nm was measured on a microplate reader.

### 4.14. Statistical Analysis

All the data used were normally distributed. The difference between groups (>2 two groups) were performed using one-way ANOVA (Tukey’s multiple comparisons test) with IBM SPSS Statistics 26 (IBM Corp., Armonk, NY, USA). All data are represented as mean ± SD, unless otherwise specified; * *p* < 0.05, ** *p* < 0.01, *** *p* < 0.001.

## 5. Conclusions

In conclusion, we have established a novel integrated strategy of in silico docking and DARTS prediction to efficiently identify the direct targets of Kae. Src was successfully identified and validated as a direct target of Kae, and we further verified its cardioprotective effect with in vitro and in vivo study. This study suggests that our strategy is convenient and efficient, and Kae might be a new potent cardioprotective drug candidate or a lead compound.

## Figures and Tables

**Table 1:** Top 10 target candidates of Kae identified by PharmMapper.

Rank	Target	Z-Score	Name	Mass (Da)
1	PDE4D	4.59774	cAMP-specific 3,5-cyclic phosphodiesterase 4D	91,115
2	HCK	4.43052	Tyrosine-protein kinase HCK	59,600
3	Cdk6	4.36472	Cell division protein kinase 6	36,938
4	PDE5A	3.82585	cGMP-specific 3,5-cyclic phosphodiesterase	99,985
5	AR	3.29088	Androgen receptor	99,188
6	ESR1	3.27299	Estrogen receptor	66,216
7	SRC	3.26386	Proto-oncogene tyrosine-protein kinase Src	59,835
8	KDR	3.12717	VEGFR2 kinase	151,527
9	CCNA2	3.07088	Cyclin-A2	48,551
10	NT5M	2.97835	5(3)-deoxyribonucleotidase, mitochondrial	25,862

**Table 2:** Target candidates of Kae identified by TargetHunter.

Number.	Score	Name	Mass (Da)	Target
CHEMBL116051	0.74	Tyrosine-protein kinase LCK	58,001	LCK
		Epidermal growth factor receptor erbB1	134,277	EGFR
		Tyrosine-protein kinase Src	59,835	SRC
CHEMBL115102	0.71	Tyrosine-protein kinase LCK	58,001	LCK
		Epidermal growth factor receptor erbB1	134,277	EGFR
		Tyrosine-protein kinase Src	59,835	SRC

## Data Availability

The data generated during the current study are available with the corresponding author on reasonable request.

## Supplementary Materials

The following are available online at https://www.mdpi.com/article/10.3390/ijms222312908/s1.
